# Four novel mutations identification in 17 beta-hydroxysteroid dehydrogenase-3 deficiency and our clinical experience: possible benefits of early treatment

**DOI:** 10.3389/fendo.2023.1267967

**Published:** 2024-02-15

**Authors:** Yunpeng Wang, Yu Xu, Huijiao Zhang, Danyang Yin, Yiming Pan, Xiwen He, Shuaiting Li, Zhi Cheng, Gaohui Zhu, Ting Zhao, Huizhe Huang, Min Zhu

**Affiliations:** ^1^ Department of Endocrine and Metabolic Diseases Children’s Hospital of Chongqing Medical University, National Clinical Research Center for Child Health and Disorders, Ministry of Education Key Laboratory of Child Development and Disorders, Chongqing, China; ^2^ Chongqing Key Laboratory of Pediatrics, Children's Hospital of Chongqing Medical University, Chongqing, China; ^3^ Office of Academic Research, The Second Affiliated Hospital of Chongqing Medical University, Chongqing, China; ^4^ Laboratory of Developmental Biology, Department of Cell Biology and Genetics, School of Basic Medical Sciences, Chongqing Medical University, Chongqing, China; ^5^ Chongqing College of Humanities, Science and Technology, Chongqing, China; ^6^ Department of Pathology, Beijing Friendship Hospital, Capital Medical University, Beijing, China; ^7^ School of Life Sciences and Technology, ShanghaiTech University, Shanghai, China

**Keywords:** 17β-HSD3 deficiency, HSD17B3, mutation, malignancy risk, androgen, testosterone

## Abstract

**Introduction:**

Individuals with 17-beta-hydroxysteroid dehydrogenase type 3 (17β-HSD3) deficiency face a multitude of challenges, primarily concerning genital appearance, potential malignancy risks, and fertility issues. This study reports our findings from an investigation involving five individuals affected by 17β-HSD3 deficiency, ranging in age from pre-adolescence to adolescence. Notably, we identified four previously unreported mutations in these subjects.

**Methods:**

Our study included a comprehensive evaluation to determine the potential occurrence of testicular tumors. The methods involved clinical examinations, genetic testing, hormone profiling, and patient history assessments. We closely monitored the progress of the study subjects throughout their treatment.

**Results:**

The results of this evaluation conclusively ruled out the presence of testicular tumors among our study subjects. Moreover, four of these individuals successfully underwent gender transition. Furthermore, we observed significant improvements in genital appearance following testosterone treatment, particularly among patients in the younger age groups who received appropriate treatment interventions.

**Discussion:**

These findings underscore the critical importance of early intervention in addressing concerns related to genital appearance, based on our extensive clinical experience and assessments. In summary, our study provides insights into the clinical aspects of 17β-HSD3 deficiency, emphasizing the vital significance of early intervention in addressing genital appearance concerns. This recommendation is supported by our comprehensive clinical assessments and experience.

## Introduction

17-beta-hydroxysteroid dehydrogenase type 3 (17β-HSD3) deficiency is an autosomal recessive disorder of sex development (DSD) that profoundly impacts sexual development (1). According to a European multi-center study, 17β-HSD3 deficiency constitutes approximately 4% of all cases of 46, XY DSD (2). The underlying cause of 17β-HSD3 deficiency lies in mutations within the HSD17B3 gene, located on chromosome 9q22.32 (3). This gene encodes a 310-amino acid protein spanning 11 exons and 10 introns (3). To date, a total of 70 distinct HSD17B3 mutations have been identified, encompassing various types such as missense mutations (the most prevalent), splice-site mutations, minor deletions, and insertions, among others (4). These mutations invariably result in the loss of functionality in the 17β-HSD3 protein, which plays a vital role in the production of sex hormones critical for male sexual development and reproduction (5).

17β-HSD3, a pivotal enzyme involved in the final step of testosterone production, is predominantly expressed in the testes (6). Its primary role is to convert Δ4-androstenedione (AND), a less biologically active androgen, into testosterone (T), a more biologically active androgen, with the assistance of the cofactor nicotinamide adenine dinucleotide phosphate (NADPH) (7, 8). In individuals with 46, XY DSD and 17β-HSD3 deficiency, it is common to observe female external genitalia, often with clitoromegaly, microphallus, and varying degrees of sex ambiguity (9). These malformations arise due to reduced production of the key androgen, testosterone (T). During fetal development, T can undergo enzymatic conversion into 5α-dihydrotestosterone (DHT) with the assistance of the enzyme 5α-reductase type 2 (SRD5A2) (10). The synthesis of both T and DHT plays a critical role in orchestrating the proper development of male genitalia during embryogenesis, with T driving the masculinization of the Wolffian ducts, seminal vesicles, and vas deferens, while DHT primarily promotes prostate growth and the development of external male genitalia (11, 12).

While hormone replacement therapy has traditionally served as the standard treatment for 17β-HSD3 deficiency, a comprehensive evaluation is imperative before commencing therapy. This evaluation should consider factors such as the risk of gonadal tumors and fertility potential (13).

To deepen our understanding of 17β-HSD3 deficiency, we conducted a comprehensive investigation encompassing molecular genetics, clinical course analysis, testis histology, immunohistochemistry (IHC), and treatment outcomes in five cases at our clinic. Our study yielded the discovery of four new HSD17B3 mutations by whole exome sequencing (WES), resulting in disruptions to testosterone synthesis. Furthermore, we provided valuable data regarding the assessment of testis tumor risk and the effects of therapeutic interventions. Our findings contribute fresh insights and evidence for the management of patients affected by 17β-HSD3 deficiency.

## Materials and methods

### Clinical studies

From June 2014 to December 2022, 5 patients with 17β-HSD3 deficiency were enrolled in this study. The patients were diagnosed based on the clinical manifestations (clinical examinations, hormonal values and karyotype analysis) and genetic characterizations. Genital phenotype was scored using the external masculinization score (EMS) (14). Whole blood samples were collected for genetic analysis. The referral age ranged from 2 years old to the puberty stage. Prepubertal patients presented with inguinal masses and clitoris enlargement, and pubertal patients presented with progressive virilization. Human choriogonadotropin hormone (hCG) stimulation tests (in addition to P1) (hCG 1,500 IU administered by intramuscular injection every other day 6 consecutive times) and gonadotropin releasing hormone (GnRH) stimulation tests (gonadorelin 2.5 μg/kg, max 100 μg) were performed.

### Hormonal testing

Estradial (E2), Dehydroepiandrosterone (DHEA), Androgen (AND), total testosterone (T), luteinizing hormone (LH) and Follicle-stimulating hormone (FSH) levels were measured using commercial kits by chemiluminescent method (Diasorin S.P.A XL-2210). Dihydrotestosterone (DHT) was measured using commercial kits by Enzyme Linked Immunosorbent assay (ELISA) method and then was detected by a Microplate Reader (Tecan Infinite E Plex).

### Genetic analysis

Genomic DNA extraction from the patients’ and their parents’ whole blood was performed using DNA isolation kits (Tiangen, Beijing, China). In total, 1 μg genomic DNA was prepared for Illumina library construction (Illumina, San Diego, CA, United States). Then, all exons and each 30 bp upstream and downstream intron were captured. Finally, an Illumina NextSeq500 high-throughput sequencer was used for the sequencing of the enriched libraries.

Clean reads were obtained after low-quality reads and adaptor sequence filtering. Then, the UCSC hg19 human reference genome was applied for clean read mapping. For further analysis, GATK software (https://software.broadinstitute.org/gatk) was used for single nucleotide polymorphism (SNP) and indel detection, and the functional influences of nonsynonymous mutations were forecasted by Polyphen2 and SIFT. Finally, dbSNP (http://www.ncbi.nlm.nih.gov/snp/), the 1,000 Genomes Project (https://www.internationalgenome.org/), the HGMD Professional (http://www.hgmd.cf.ac.uk/ac/index.php) databases and Sanger sequencing were used for mutation novelty confirmation. 17β-HSD3 conservation analysis was performed by UniProt (http://www.uniprot.org/UniProt/).

### *In silico* analysis and molecular modeling

Two online software programs, SIFT (http://sift.jcvi.org/) and PolyPhen-2 (http://genetics.bwh.harvard.edu/pph2/), were used to assess the pathogenicity of new mutations. Homology of the mutated nucleotide was analyzed with National Center for Biotechnology Information Website BLAST Tools. UniProt (https://www.UniProt.org/) was employed for evolutionary assessment. The homology model was executed using Modeler10.1 software. The RCSB PDB database (PDB code: 5GT9) was utilized to obtain a 3D templet protein structure based on water molecules, ions, heteroatoms and elimination of all ligands. A 3D homology model and mutation protein structure were generated by single templet modeling and pymol2.2, respectively. Finally, the protonation states of the wild-type and mutated proteins were determined using an H++ server. Additionally, all missing hydrogen atoms were inserted by pymol22, and 3D figures were generated by pymol2.2.

### Hematoxylin and eosin staining and IHC

Testis tissues from 4 patients (Patient 2 – Patient 5) were collected for histological analysis. Ten percent neutral buffered formalin (CAS: 3046-49-9, 10%, Sigma−Aldrich) and Bouin fixative (Sigma−Aldrich) were used for sample fixation for 24 hours. After fixation, these testis samples were dehydrated, paraffin embedded and cut into 5 μm thick sections. The sample sections were then deparaffinized and rehydrated for HE and IHC staining. For IHC, tissue sections were boiled in 10 mM citrate buffer for 30 mins to retrieve antigen. Goat serum was utilized for nonspecific binding site blocking. Tissue sections were incubated with the primary antibodies (4°C, overnight). Then, 3% H2O2 was applied for endogenous peroxidase blocking. The sections were subsequently washed and incubated with the secondary antibody (37°C, 30 mins) and counterstained with hematoxylin. The primary antibodies utilized for IHC were as follows: α-SMA (Cat No. 14395-1-AP, dilution 1:1500, Proteintech), Mage-4 (Cat No. 12508-1-AP, dilution 1:200, Proteintech), AFP (Cat No. 14550-1-AP, dilution 1:100, Proteintech), AR (Cat No. 22089-1-AP, dilution 1:200, Proteintech), HCG (Cat No. 25014-1-AP, dilution 1:1000, Proteintech), CD117 (Cat No. 18696-1-AP, dilution 1:100, Proteintech) and PLAP (Cat No. 18507-1-AP, dilution 1:100, Proteintech). The secondary antibody was (Cat No. SA00001-2, dilution 1:1000, Proteintech). The results were analyzed using a Leica fluorescence stereomicroscope (Leica, DM6B).

### Plasmid construction, cell culture and transient transfection

The 17β-HSD3 sequence contained in the pcDNA3 plasmid was designed with a C-terminal FLAG epitope tag (17β-HSD3-FLAG) and constructed by VectorBuilder. In short, the template originated from this plasmid, and Pfu Polymerase (Promega, Madison, WI, USA) was employed for HSD17B3 mutation construction, which led to the 17β-HSD3 protein mutations p. P228L, p. P191S, p. Y198F, and p. L239R. All the constructed mutations were confirmed by Sanger sequencing. Human embryonic kidney (HEK) 293T cells were utilized for enzyme measurement. The cell line was derived from the Cell Bank of the Chinese Academy of Sciences (Shanghai, China). The mixed nutrient solution contained Dulbecco’s modified Eagle medium (DMEM), fetal bovine serum (10%), glutamine (2 mM), penicillin (50 units/mL) and streptomycin (50 μg/ml) (Sigma−Aldrich, St. Louis, Missouri) was used for cell culture. Cells were cultured at 37°C and 5% CO2. A total of 10^6^ HEK-293T cells were seeded per well in 6-well plates. Twenty-four hours later, Lipofectamine 3000 (Invitrogen, Carlsbad, CA, United States) was employed for transient transfection following the manufacturer’s instructions.

### Quantitative real-time-PCR analysis and Western blot

PCR experiments were performed in Patient 1 and Patient 2 families targeting exon 1 of HSD17B3. Each patient’s cDNA was first amplified using ChamQ Universal SYBR qPCR Master Mix (Vazyme, China) in accordance with the manufacturer’s directions. Primer-BLAST was used to design primers (**Table S1**). A denaturation step at 95°C for 3 min was followed by 39 cycles at 95°C for 10 s and 60°C for 30 s to set up the conditions for amplification. The mRNA expression levels of the target genes were determined by the 2^–ΔΔCt^ technique, and the housekeeping gene gapdh was applied as the internal control. For western blotting, 48 hours after successful plasmid transfection, cells in each well were lysed in 100 μL RIPA buffer (Sigma−Aldrich) containing a protease inhibitor cocktail (Roche, Basel, Switzerland). Then, the collected samples were centrifuged at 15,000 × g for 15 min at 4°C for debris removal. Twenty micrograms of total protein were transferred to nitrocellulose membranes after protein concentration determination by a PierceTM BCA protein assay kit (Thermo Scientific, Rockford, IL), followed by blocking in 5% nonfat milk and incubation at 4°C for 12 hours with primary anti-FLAG M2 antibody. The primary antibodies used in this experiment were as follows: FLAG M2 antibody (F1804, dilution 1:1000, Sigma−Aldrich) and β-Actin rabbit monoclonal antibody (15G5A11/E2) (Cat MA1-140, dilution 1:5000, Proteintech). The secondary antibody was HRP-conjugated AffiniPure goat anti-mouse IgG (H + L) (Cat No. SA00012-1, dilution 1:2000, Proteintech).

### Enzyme activity assay

HEK-293T transfected cells were employed for enzyme function analysis. The transfected cells were cultivated for 48 h at 37°C (3 mL per well), subsequently the cells were collected (300 × g, 10 min) and ultrasonically cleaved. And then they were resuspended to charcoal treated serum free medium (200 μL) containing 200 nmol AD and 500 μM NADPH. Reactions were stopped at 30 min by adding 20 μL methanol. Subsequently, culture solutions were immediately collected and centrifuged at 3000 rpm for 10 mins at 4°C to obtain the supernatants for testosterone measurement by ELISA method following the manufacturer’s instructions (Wuhan Fine Biotech Co., Ltd.) using a Microplate Reader (Tecan Infinite E Plex).

### Quantification and statistical analysis

The statistical analyses were performed using Prism 5.01 (GraphPad): Tukey’s post-test. Posttest p values are as follows: ∗∗∗∗p < 0.0001.

## Results

### Clinical, genetic characterizations and treatment of patients with 17β-HSD3 deficiency

#### Patient 1

A 6-year-old female patient was admitted to our hospital following the extraction of a right inguinal mass. She presented with typical female external genitalia, was raised as a female, and exhibited a squatting urination pattern. The parents were not consanguineous, and there was no family history of infertility. Physical examination revealed the absence of breast development, infantile female genitalia, a diminutive clitoris, and discernible urethral and vaginal openings. Notably, there were no palpable masses in the bilateral groin region. The EMS score was recorded as 0.5. The parents advocated for female gender assignment, and consequently, no medical treatment was administered.

#### Patient 2

At birth, the external genitalia of this child were initially classified as female, and the child was accordingly raised as such. However, at the age of 3, the child was referred to a local hospital due to the presence of a groin mass, which was subsequently identified as testicular tissue through B-ultrasound examination. At age 7, chromosomal karyotype analysis revealed a male pattern (46, XY). Remarkably, by age 10, the child exhibited clitoral hypertrophy measuring approximately 1.8 cm in length, as well as sparse pubic hair. On physical examination, bilateral subcutaneous masses resembling “labia majora” were palpable, each with a volume of approximately 2.0 ml. Both the urethra and vagina were visible in the perineum, and the EMS score was recorded as 3.

Subsequent to these findings, the child’s gender was redefined as male, and surgical correction for existing hypospadias was performed. Hormone therapy with testosterone undecanoate commenced at age 11. At the last follow-up visit at age 13 in September, the penis had grown to a length of 4.0 cm with a circumference of 5.5 cm, although urination still occurred in a squatting position.

#### Patient 3

This patient was born with external genitalia resembling the female vulva and was initially raised as female. However, the patient was later referred to our hospital due to clitoral enlargement, voice deepening, and an inguinal hernia development.

Physical examination revealed evidence of pubic and axillary hair growth but no Adam’s apple or breast development. The penis measured approximately 4.0 cm in length with a diameter of about 1.0 cm, and the urethral opening was located at the ventral base of the penis. Additionally, a visible vaginal opening was observed in the perineum. Pubic hair growth was noted at the base of the penis and pubic symphysis, with an estimated testicular volume of around 2.0 ml. The EMS score was recorded as 5. Consequently, the decision was made to transition to male gender, and treatment with testosterone undecanoate was initiated.

As a result of this therapy, the penis grew to a length of 4.5 cm with a circumference of 5.0 cm, and testicular volume increased to 6 ml.

#### Patients 4 and 5

Patients 4 and 5, who were twins, were initially raised as females but exhibited clitoral hypertrophy shortly after birth, which became more prominent by age 1. Both had undescended testes as seen on ultrasound. After surgically placing the testes in the scrotum, they were referred to our hospital’s endocrinology department.

Upon examination, Patient 4 had a 2.5 cm penis with both urethral and vaginal openings at the base. Patient 5 had a 2.3 cm penis with only a urethral opening at the base. Their scrotums were underdeveloped, and their testes measured about 2.0 ml each, resulting in an EMS score of 6. Both patients transitioned to male gender. After testosterone undecanoate treatment and surgery, Patient 4’s penis grew to 4.0 cm, with a testicular volume of 3.0 ml. Patient 5’s penis reached 3.9 cm, with a testicular volume of 2.5 ml.

**Figure 1:**
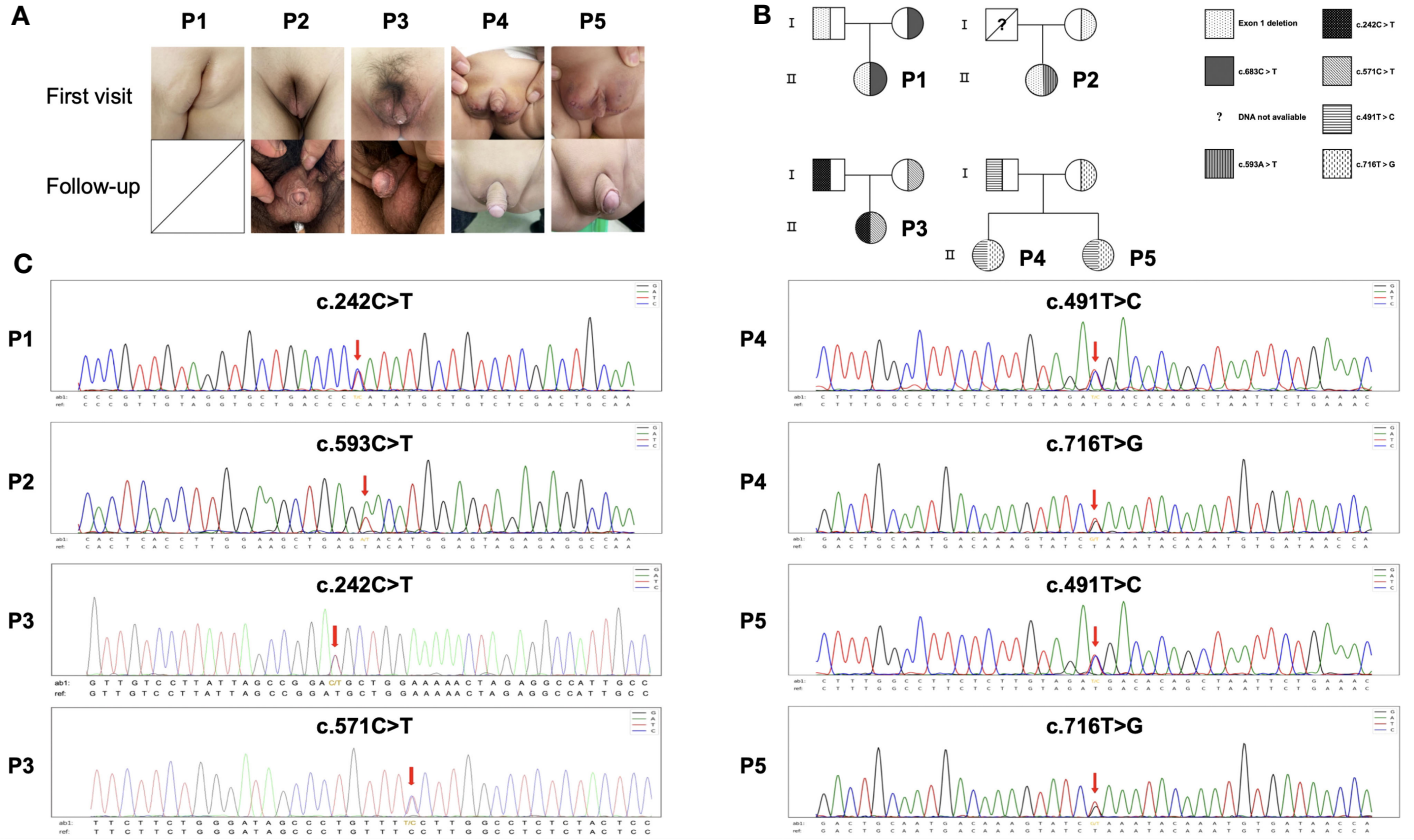
Pedigree, missense mutations and clinical manifestations of HSD17B3 deficiency patients. **(A)** Genitalia appearances of patients at first visit and following-up. **(B)** Family pedigree. Unavailable information of deceased father of Patient 2. **(C)** HSD17B3 DNA sequencing of the patients; the mutation site in HSD17B3 is marked with an arrow.

Representative figures of these 5 patients at initial diagnosis and after treatment (Patient 1 did not reach the follow-up period) are presented in **Figure 1A**. Chromosomal karyotypes were confirmed as 46, XY, and no uterine or ovarian structures were detected in these patients. Patients 1-3 were born with fully developed female genitalia, while Patients 4 and 5 were born with clitoral hypertrophy of the external genitalia and urethral vaginal openings. All patients were initially raised as females and subsequently exhibited progressive virilization. EMS scores were extensive, and overall clinical phenotypes ranged from moderate to severe (EMS score ≤6).

Hormone levels were examined in all five patients. AND accumulated in adolescent Patients 2 and 3, with significantly decreased T/AND baselines. In preadolescent patients, the T/AND ratio could not be calculated due to low values of T and AND under the unactivated hypothalamic-pituitary-gonad axis, necessitating further evaluation via the HCG excitation test. Two preadolescent children (Patients 4 and 5) completed the HCG excitation test. Patient 4 exhibited normal testosterone synthesis after the HCG stimulation test but a decreased T/AND ratio, indicating relative defects in testosterone synthesis. The other patient displayed insufficient testosterone synthesis after the HCG stimulation test, suggesting functional defects of testicular interstitial cells. In the GnRH excitation test, LH and FSH peaks significantly increased in all five children, surpassing normal responses and suggesting hypergonadotropic hypogonadism. Detailed clinical findings for the patients at initial diagnosis and follow-up are listed in **Tables 1**, **2**, respectively.

**Table 1:** Clinical manifestations of 5 patients with 17-βHSD3 deficiency at initial diagnosis.

	Patient 1	Patient 2	Patient 3	Patient 4	Patient 5
First visit	6 years	11years	10 years	2 years	2 years
Follow-up	–	13 years	14 years	4 years	4 years
Referral reason	Virilization	Virilization	Virilization	Virilization	Virilization
External genital phenotype at birth	Female external genitalia	Female external genitalia	Female external genitalia	Ambiguous genitalia, clitoromegaly	Ambiguous genitalia, clitoromegaly
Rearing sex	F	F	F	F	F
Height (cm)	112.5	150.0	155.5	–	–
Weight (kg)	24.0	56.5	53.5	10.5	10.0
Karyotype	46, XY	46, XY	46, XY	46, XY	46, XY
Phallic length (cm)	0.1	1.8	4.0	2.5	2.3
Testes’ locations	Left lower quadrant, right orchiectomy	Inguinal region	Inguinal region	Scrotum	Scrotum
Testes size (cm) (left, right)	-, 1.6×0.8×0.6	2.65×1.50×0.80, 2.2×1.30×0.70	3.0×1.9×1.1, 3.0×1.9×1.1	1.7×0.9×0.6, 1.9×1.0×0.8	1.6×0.9×0.6, 1.8×0.9×0.7
Vaginal and urethral openings	Separate	Separate	Separate	Single	Single
Masculinization score	0.5	3	5	6	6
HSD17B3 mutation	c.683C>T, Exon1 deletion	c.593A>T, Exon1 deletion	c.242C>T, c.571C>T	c.491T>C, c.716T>G,	c.491T>C, c.716T>G,
E2 pmol/ml	<73.4 (<73.4)	179 (<146.8)	<73.4 (<146.8)	<73.4 (<73.4)	<73.4 (<73.4)
DHEA (umol/l)	<0.41 (<1.02)	2.59 (<4.15)	1.380 (<4.15)	<0.41 (<0.41)	<0.41 (<0.41)
AND (nmol/l)	<1.05 (<3.92)	4.17 (<4.9)	2.55 (<4.9)	<1.05 (<1.05)	<1.05 (<1.05)
T (nmol/l)	<0.69 (<0.69)	<0.69 (<0.72)	0.87 (<1.87)	<0.09 (<0.69)	<0.69 (<0.69)
LH (mIU/mL)	<0.10 (0.12-0.2)	10.3 (0.2-3.4)	5.11 (0.2-3.4)	<0.10 (0.12-0.2)	0.2 (0.12-0.2)
FSH (mIU/mL)	4.71 (1.1-4.3)	12 (0.51-5.00)	19.70 (0.51-5)	0.36 (1.1-4.3)	1.73 (1.1-4.3)
T/AND		<0.17	0.34		
DHT (pg/ml)				17.84 / <50 (40.5-355 / ≤50)	19.12 / <50 (40.5-355 / ≤50)
LH basal level (mIU/mL)	<0.10	10.00	5.10	1.22	1.2
FSH basal level (mIU/L)	4.70	12.00	19.70	3.13	4.28
LH after GnRH (30min) (mIU/L)	3.37	49.20	56.00	19.6	23.1
FSH after GnRH (30min) (mIU/mL)	25.70	16.20	30.50	7.72	11.8
LH after GnRH (60min) (mIU/mL)	2.45	43.50	43.80	13	14.3
FSH after GnRH (60min) (mIU/mL)	26.90	16.50	32.70	9.83	12.9
T basal level (nmol/l)			0.87	<0.69	<0.69
T after HCG (nmol/l)			13.2	11.2	5.51
AND basal level (nmol/l)			2.55	<1.05	<1.05
AND after HCG (nmol/l)				6.9	2.89
T/AND basal level			0.34		
T/AND after HCG				1.62	1.94

**Table 2:** Sex hormone of 5 patients with 17-βHSD3 deficiency at following-up.

	Patient 1	Patient 2	Patient 3	Patient 4	Patient 5
E2 pmol/ml		67.8 (<158)	60.3 (<158)	<59.5 (<108)	<59.5 (<108)
DHEA (umol/l)		5.7 (0.55-3.37)	7.33 (0.55-3.37)	<0.14 (<0.14)	<0.14 (<0.14)
AND (nmol/l)		26.8 (1.41-5.5)	12 (1.41-5.5)	<1.05 (<1.05)	<1.05 (<1.05)
T (nmol/l)		4.72 (0.87-13.27)	2.68 (0.87-13.27)	<0.69 (<0.69)	<0.69 (<0.69)
LH (mIU/mL)		20.5 (0.8-10.1)	22.8 (0.8-10.1)	0.23 (0.1-0.27)	<0.10 (0.1-0.27)
FSH (mIU/mL)		39.9 (1.8-9.6)	39.5 (1.8-9.6)	1.72 (0.2-0.85)	0.78 (0.2-0.85)
T/AND		0.18	0.22	<50 (≤50)	<50 (≤50)
T basal level (nmol/l)		4.72	2.68	<0.69	<0.69
T after HCG (nmol/l)		5.62	11.8	9.19	4.4
AND basal level (nmol/l)		26.8	12.3	<1.05	<1.05
AND after HCG (nmol/l)		27.10	15.2	5.88	2.61
DHT basal level (pg/ml)		531.73			
DHT after HCG (pg/ml)		739.52			
T/AND basal level		0.18	0.21	–	–
T/AND after HCG		0.21	0.78	1.56	1.68

### Detection of HSD17B3 mutations in 5 patients

Sequence analysis of the HSD17B3 gene unveiled heterozygous mutations in all patients. Except for the deceased father of Patient 2, all other parents were confirmed to be carriers (**Figures 1B, C**). In Patients 1 and 2, heterozygous missense variants were identified: HSD17B3 c.683C>T (p. P228L) and c.593A>T (p. Y198F), respectively. Patient 3 exhibited two heterozygous missense variants, namely, c.242C>T (p. T81) and c.571C>T (p.P191S). As for Patients 4 and 5, being twins, they carried the mutations c.491T>C (p. M164T) and c.716T>G (p. L239R).

**Figure 2:**
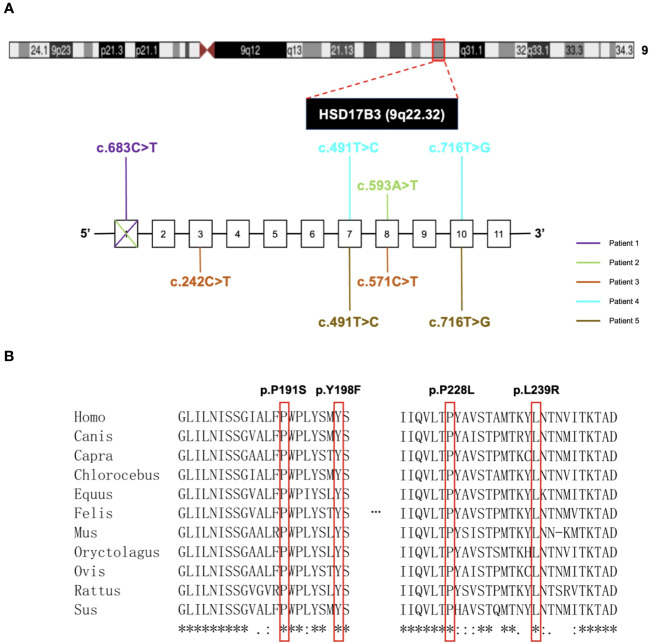
Gene location and analysis of mutations in HSD17B3 deficiency patients. **(A)** The locus for chromosomal region 9q22.32. The bottom next to the chromosomal figure showed the mutations conclusion. **(B)** Conservation analysis of 4 novel-mutated residues across eleven species.

Furthermore, exon 1 deletion was observed in Patient 1 and 2 (**Figure S1**). Quantitative PCR (qPCR) results indicated that nearly 40% of exon 1 expression was detected in both Patient 1 and Patient 2. Detailed mutational information for the patients is provided in **Figure 2A**. In total, our study identified four previously unreported mutations, namely, p. P191S, p. Y198F, p. P228L, and p. L239R.

To assess pathogenicity, we employed PolyPhen-2 and SIFT for prediction. Notably, p. P191S, p. Y198F, and p. P228L mutations were predicted to be damaging, while the p. L239R mutation was deemed less likely to induce damage (**Table 3**). It is worth mentioning that all mutation sites demonstrated a high degree of conservation among different species, as illustrated in **Figure 2B**.

**Table 3:** Predictions of four new mutations of HSD17B3.

	SIFT		PolyPhen-2			
	Score	Prediction	Sensitivity	Specificity	Score	Prediction
p. P228L	-9.128	Deleterious	0.00	1.0	1.000	Probably damaging
p. Y198F	-3.719	Deleterious	0.00	1.0	1.000	Probably damaging
p. P191S	-7.239	Deleterious	0.62	0.92	0.961	Probably damaging
p. L239R	0.651	Neutral	0.95	0.53	0.014	Benign

In our investigation, we constructed mutation variants of 17β-HSD3 targeting four critical amino acids to investigate potential changes in enzyme activity. The wild-type and mutational residues are visually represented in **Figure S2**.

### Histology and IHC of testis tissue deficient in HSD17B3

**Figure 3:**
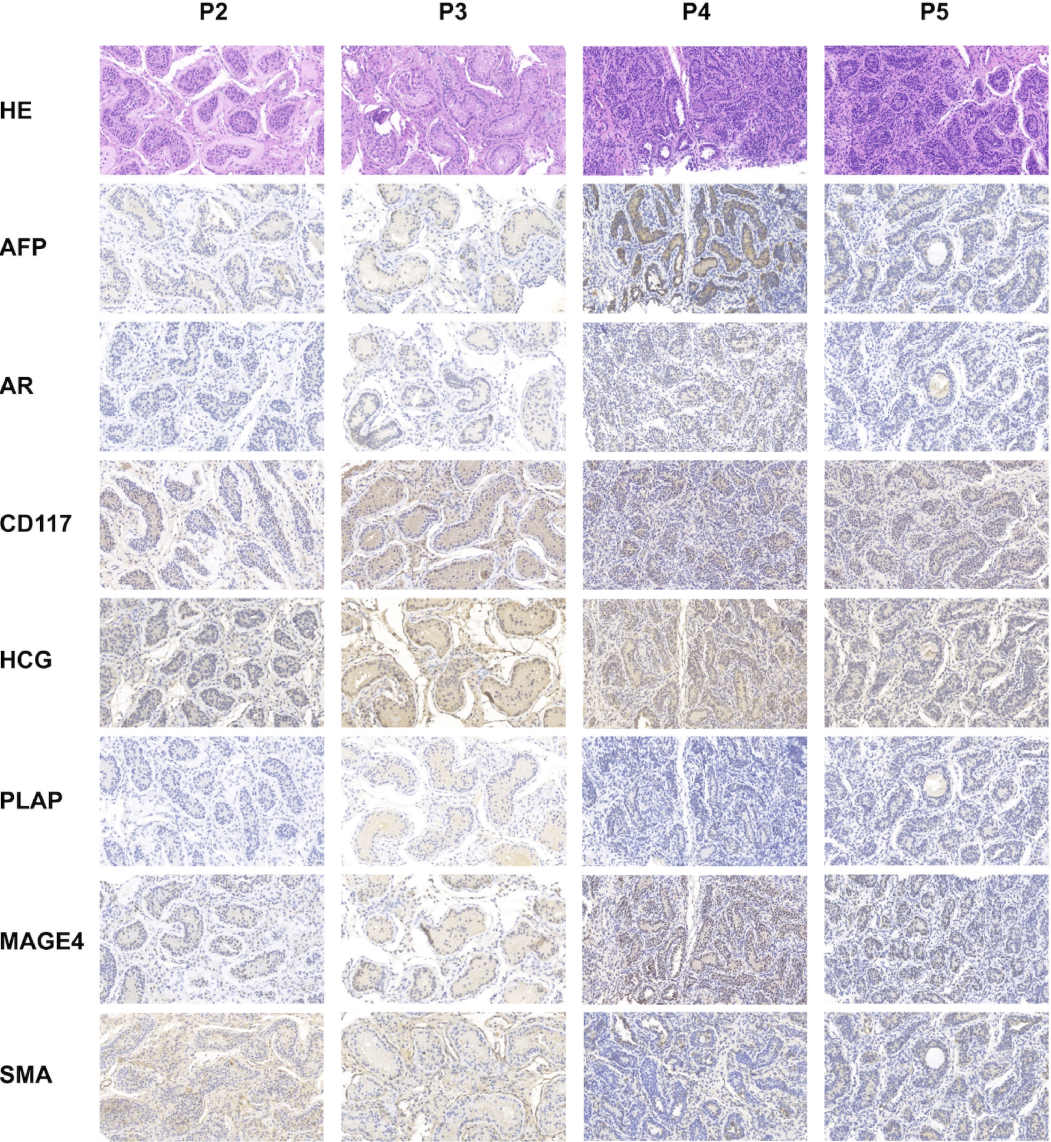
Histologic and immunohistochemical characterization of the testes deficient in HSD17B3. Testes were biopsied and surgically fixed within the scrotum before starting testosterone replacement therapy. HE and representative images of immunohistochemistry to evaluate testicular development and tumor risk. MAGE4 staining showing no obvious germ cells, Patient 2 excepted (germ cells occasionally seen). SMA staining revealing normal peritubular cells. AR staining identifying some Sertoli cells and Leydig cells. AFP, CD117, HCG and PLAP staining confirming the absence of testis malignancy.

All patients underwent orchidopexy before commencing testosterone replacement therapy. Regrettably, histopathological examination (HE) and immunohistochemistry (IHC) data could not be presented for Patient 1 due to the unavailability of a testicular section. Patient 2 exhibited a small number of scattered mature Leydig cells. Some spermatogenic tubules displayed lumens, and there was significant thickening of the basal membrane around the tubules. However, certain tubules lacked lumens, and the presence of luminal germ cells was sporadic. In the case of Patient 3, interstitial fibroplasia was observed, and mature Leydig cells were notably absent. Similar to Patient 2, some spermatogenic tubules had lumens, while most lacked lumens. Furthermore, the presence of luminal germ cells was not conspicuous. Both Patients 4 and 5 displayed undifferentiated spindle cells in the interstitium, and mature Leydig cells were absent in both cases. However, there were differences between the two patients. In Patient 4, some tubules exhibited partially dilated lumens, but no germ cells were evident within the tube wall. Conversely, in Patient 5, there were no discernible small lumens, and germ cells were absent in the luminal space. It is essential to note that no cell tumors were detected in any of the patients, suggesting a lack of malignancy risk. This conclusion was further validated by negative staining results with specific markers for these cells, as presented in **Figure 3**.

### Functional analysis of the four novel mutations

**Figure 4:**
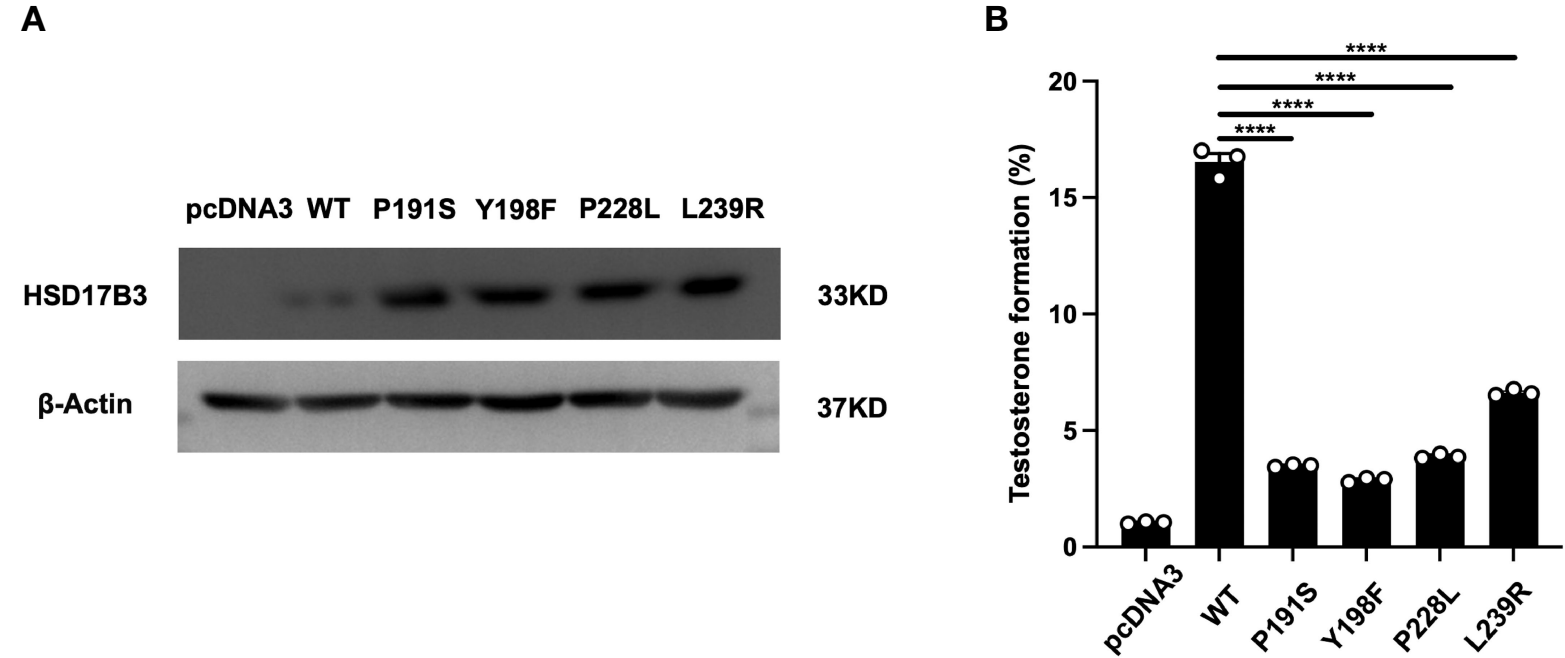
Expressions and activities of wild-type and mutant 17β-HSD3 enzymes. **(A)** Western blot of FLAG-tagged wild- type 17β-HSD3 and mutants. β-Actin was applied as the control and analyzed using an anti-β-actin antibody. **(B)** Enzyme activity of wild-type and mutants17β-HSD3. The ELISA analysis yielding the percentage of testosterone that was formed from the initially provided androstenedione. Data from three independent experiments and represented mean ± SEM. (n = 3, Means ± SEM, ****P < 0.0001).

HEK-293 T cells were subjected to transient transfection with plasmids containing wild-type 17β-HSD3 and the newly identified variants (p. P191S, p. Y198F, p. P228L, and p. L239R). Each cDNA construct was modified by incorporating a FLAG epitope tag immediately following the coding region to facilitate protein detection. Western blotting was employed to evaluate protein expression. As depicted in **Figure 4A**, the mutants and wild-type 17β-HSD3 were successfully expressed, with some mutant enzymes exhibiting even higher expression levels.

Subsequently, lysates of transfected HEK-293 cells containing wild-type 17β-HSD3 and its mutants were prepared for the assessment of enzyme activity. The wild-type enzyme converted approximately 17% of AD to T, whereas the mutants displayed significant reductions in enzyme activity (**Figure 4B**). Specifically, mutants p. Y198F exhibited only 2.88% testosterone formation. Notably, mutants p. L239R demonstrated most higher testosterone formation compared with other mutants, with values of 6.62%.

## Discussion

In this study, we thoroughly documented the clinical symptoms and genetic characteristics of five patients with 17β-HSD3 deficiency. Each patient was diagnosed with 17β-HSD3 deficiency and exhibited varying degrees of masculinization. Four novel mutations—p. P228L, p. Y198F, p. P191S, and p. L239R4—were discovered by genetic testing. Comprehensive research on testis histology was carried out, and the majority of patients received treatment. The results showed that patients in our cohort with 17β-HSD3 deficiency are not at risk for gonadal malignancies. Additionally, treatment outcomes tend to be more favorable in younger patients who receive treatment.

Hormone stimulation tests employed in diagnosing 17β-HSD3 deficiency did not consistently yield 100% accurate diagnoses. 17β-HSD3 plays a critical role in converting androstenedione (AND) to testosterone (T) (7, 8). Alterations in the structure of 17β-HSD3 can lead to partial or complete loss of enzyme activity, resulting in an accumulation of substrate AND, reduced T synthesis, and a decline in the T/AND ratio. Previous studies have suggested that a T/AND ratio below 0.8 should raise suspicion of 17β-HSD3 deficiency (1, 15, 16). This effect was also observed following an hCG administration test (1, 9, 17, 18). Laboratory evaluations conducted during puberty (in Patient 2 and 3) in our patient cohort revealed significant substrate AND accumulation, despite basal T levels within the reference range. In contrast, prepubescent patients (Patient 1, 4, and 5) exhibited low T and AND values due to inactivity of the hypothalamic-pituitary-gonadal axis. Consequently, HCG stimulation tests were performed (excluding Patient 1), revealing relative deficiencies in testosterone production for Patient 4 and testicular interstitial cell dysfunction for Patient 5, even though their T/AND ratio exceeded 0.8. Based on our findings, it is possible for patients with 17β-HSD3 deficiency to exhibit a T/AND ratio above 0.8 following an HCG test. This phenomenon is likely attributed to isoenzymes (e.g., 17β-HSD5) (9, 16, 19), residual enzyme activity (20–24), and individual variations in extra-testicular enzyme activity (9, 16, 19). Therefore, additional molecular testing is particularly crucial for confirming the diagnosis of this condition.

Patient 1 and 2 both exhibited a deletion in exon 1. Previous reports have documented patients with a similar presentation, characterized by severe male underdevelopment of the external genitalia (25). Deletion of exon 1 can lead to changes in the start codon, resulting in a loss of enzymatic activity. Additionally, in Patient 1 and 2, we identified two novel point mutations, p.P228L and p.Y198F. Computational predictions strongly suggested the deleterious nature of these mutations, a hypothesis corroborated by functional assessments conducted in our study. Patient 3 to 5 harbored rare biallelic missense mutations. Patient 3 carried c.242C>T; p.T81M and c.571C>T; p.P191S mutations in the HSD17B3 gene. Patient 4 and 5 exhibited mutations c.491T>C; p.M164T and c.716T>G; p.L239R. Among these, c.242C>T; p.T81M and c.491T>C; p.M164T mutations have been previously reported (26, 27), with the latter being confirmed to exhibit enzymatic activity at only 5% to 6.67% of wild-type 17β-HSD3 (27). Furthermore, the functionality of two additional novel mutation sites in Patient 3 to 5 was impaired, as established by our investigations. Although computational prediction algorithms suggested potential tolerance of p.L239R, our functional studies demonstrated a significant reduction in the conversion of androstenedione to testosterone compared to the wild-type enzyme. These findings collectively underscore the pathogenic disruption of 17β-HSD3 enzyme function as the underlying cause of the 46, XY DSD phenotype observed in our subjects.

Another concerning problem is gonadal tumors possibility. No aberrant development or tumor-like alterations were found, indicating that our individuals had a low predisposition for malignancy. Regarding tumor risk, the youngest patient with 17β-HSD3 deficiency and concomitant testicular tumors was 4 years old, exhibiting positive OCT3/4 staining in germ cells, suggestive of CIS lesions (28). The oldest patient with interstitial cell tumors was 61 years old (29). It is noteworthy that all three cases of malignancy in 17β-HSD3-deficient demonstrated varying degrees of interstitial cell proliferation (30–32). Particular attention should be paid to such patients, as interstitial cell proliferation may increase the risk of malignant tumors. Furthermore, careful consideration should be given to the need for prophylactic testicular removal, as the incidence of germ cell tumors in 17HSD3 deficiency syndrome is low, accounting for only 5.0% (1).

Replacement therapies involving estrogen and testosterone undecanoate are employed in female patients following gonadectomy and male patients, respectively (13). In our study, only Patient 1 opted to retain the female gender, whereas the remaining four patients underwent gender transition from female to male. Notably, younger patients, namely Patient 4 and Patient 5, exhibited more favorable treatment outcomes in terms of genital appearance compared to adolescent Patient 2 and 3. This discrepancy in treatment effects may be attributed to the milder disease phenotype at birth in Patient 4 and 5, coupled with the initiation of treatment at an earlier age. In the view of our patients, commencing testosterone undecanoate replacement therapy earlier may yield superior results in individuals with 17HSD3 deficiency opting for a male gender following comprehensive evaluation. However, given the limited treatment data, a larger patient cohort is warranted for a more precise conclusion.

## Conclusions

In summary, through thorough studies of 5 17β-HSD3-deficient individuals, we learned that 4 novel mutations, p. P228L, p. Y198F, p. P191S and p. L239R4, are functional defects in testosterone production. Moreover, due to the low risk for testicular malignancy, prophylactic testicular removal should be considered carefully. However, hormonal imbalance still needs to be considered, with estrogen and testosterone undecanoate remaining the primary choice for replacement therapy. Combined with our patient outcomes, earlier intervention is necessary once the disease is diagnosed and the gender role is decided, as this may bring better external genital appearance and make the sex-changing individuals adapt to new life earlier.

## Data availability statement

The data presented in the study are deposited at the National Genomics Data Center, Beijing Institute of Genomics, Chinese Academy of Sciences, and China National Center for Bioinformation under the accession number GVM000639. (https://bigd.big.ac.cn/gvm/getProjectDetail?Project=GVM000639).

## Ethics statement

The studies involving humans were approved by Biomedical Ethics Committee, Children’s Hospital Affiliated to Chongqing Medical University. The studies were conducted in accordance with the local legislation and institutional requirements. The participants provided their written informed consent to participate in this study. The manuscript presents research on animals that do not require ethical approval for their study. Written informed consent was obtained from the individual(s), and minor(s)’ legal guardian/next of kin, for the publication of any potentially identifiable images or data included in this article.

## Author contributions

MZ: Funding acquisition, Resources, Supervision, Writing – review & editing. YW: Conceptualization, Formal Analysis, Investigation, Methodology, Resources, Writing – original draft, Writing – review & editing. YX: Conceptualization, Data curation, Formal Analysis, Investigation, Methodology, Writing – review & editing, Writing – original draft. HZ: Data curation, Writing – review & editing. DY: Data curation, Writing – review & editing. YP: Data curation, Writing – review & editing. XH: Data curation, Writing – review & editing. SL: Data curation, Writing – review & editing. ZC: Data curation, Writing – review & editing. GZ: Data curation, Writing – review & editing. TZ: Data curation, Writing – review & editing. HH: Data curation, Resources, Supervision, Writing – review & editing.

## Supplementary material

Supplementary Figure 1 Transcriptional profiles of HSD17B3 exon 1 for Patient 1 and 2. Two different primers for HSD17B3 exon 1 expression detection in Patient 1 **(A)** and 2 **(B)**.

Supplementary Figure 2 Predicted crystal structural models of the wild-type and mutants. **(A-E)** Crystal structures of wild-type and mutant human HSD17B3.
