# Supplementary figures and images for: Four novel mutations identification in 17 beta-hydroxysteroid dehydrogenase-3 deficiency and our clinical experience: possible benefits of early treatment
Source: Front Endocrinol (Lausanne). 2024 Feb 15;14:1267967. doi: 10.3389/fendo.2023.1267967 (PMC10902039; doi:10.3389/fendo.2023.1267967)

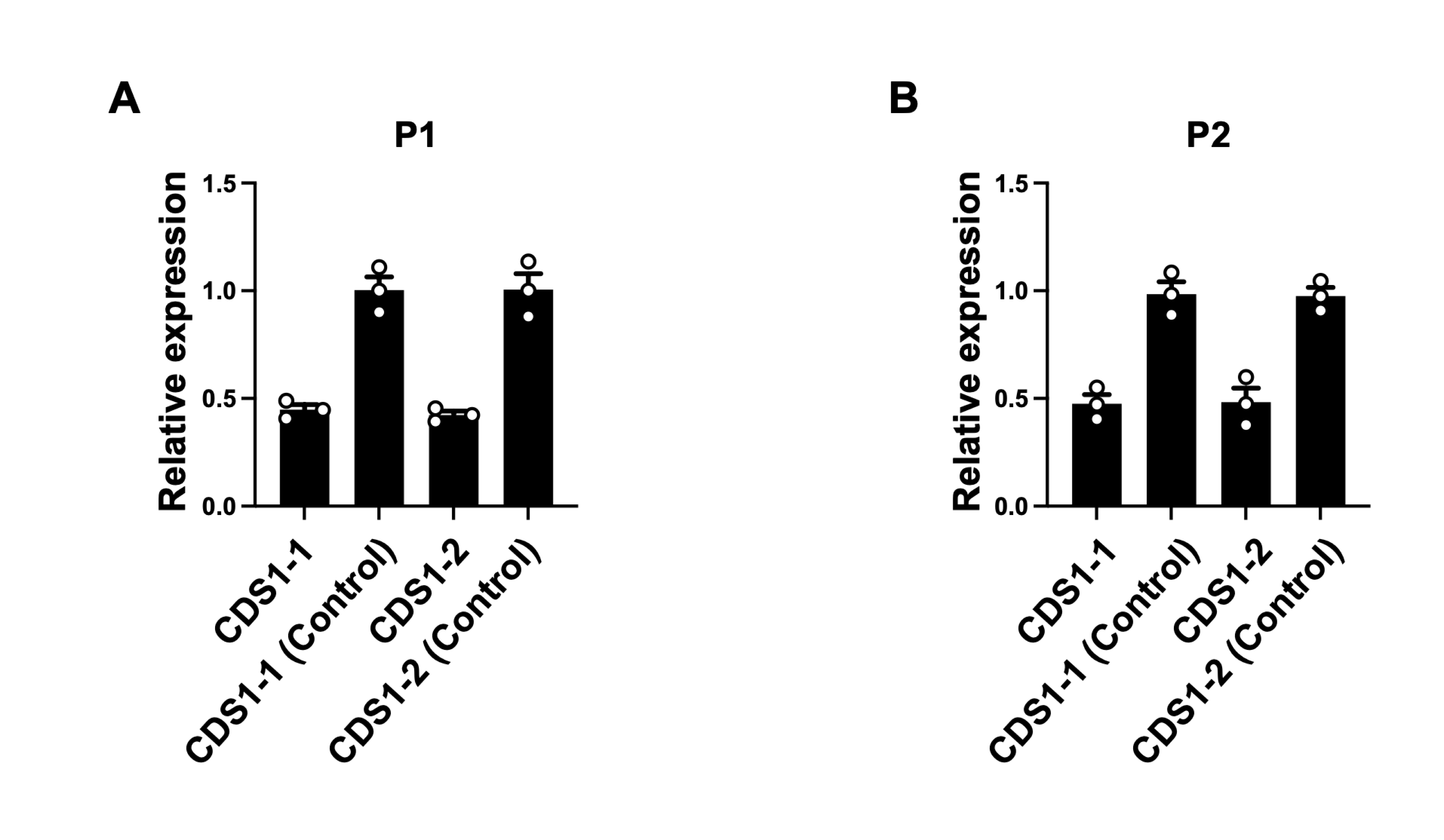

Supplement: Supplementary Figure 1 — Transcriptional profiles of HSD17B3 exon 1 for Patient 1 and 2. Two different primers for HSD17B3 exon 1 expression detection in Patient 1 (A) and 2 (B). [file Image_1.tif]

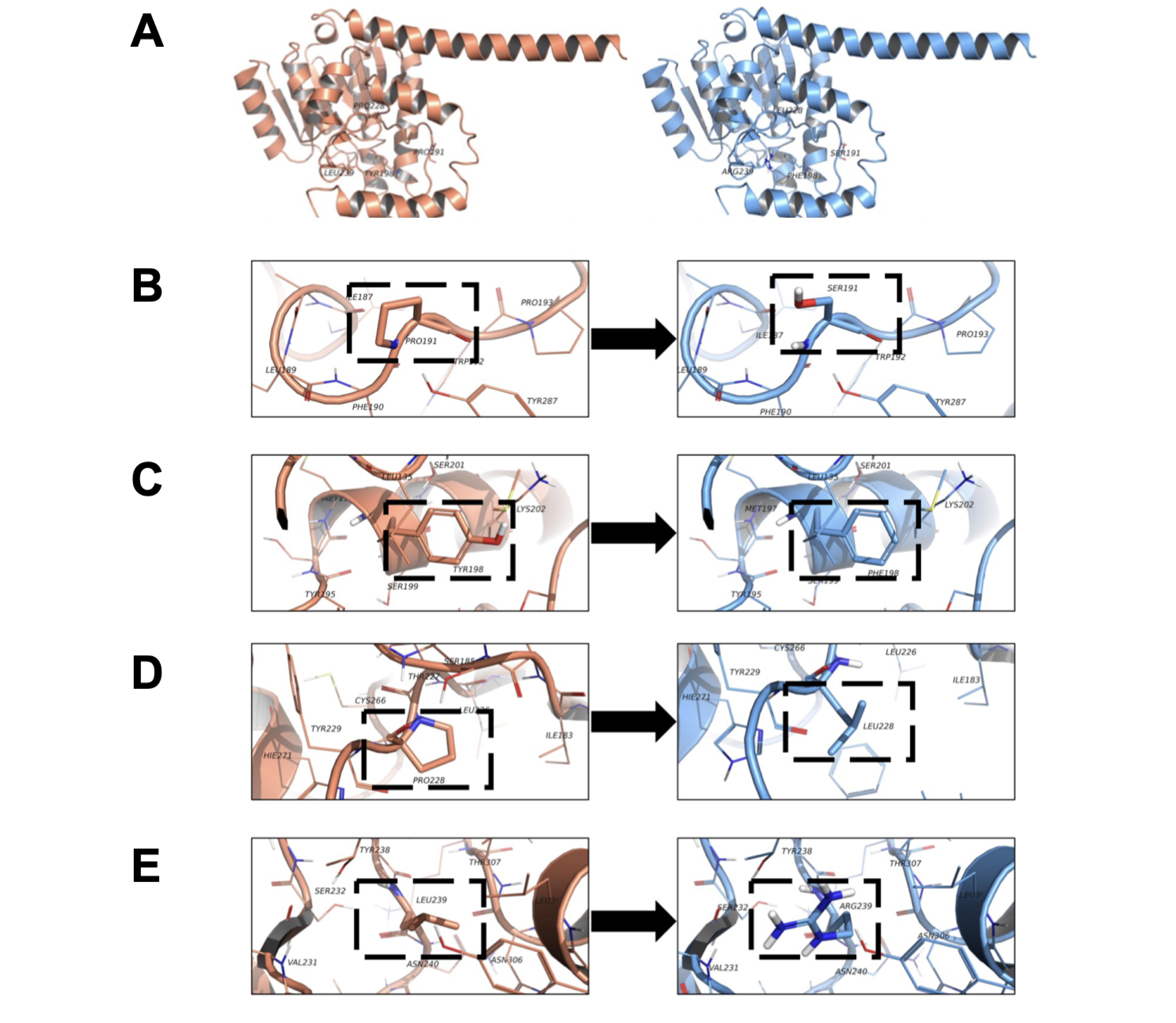

Supplement: Supplementary Figure 2 — Predicted crystal structural models of the wild-type and mutants. (A-E) Crystal structures of wild-type and mutant human HSD17B3. [file Image_2.tif]

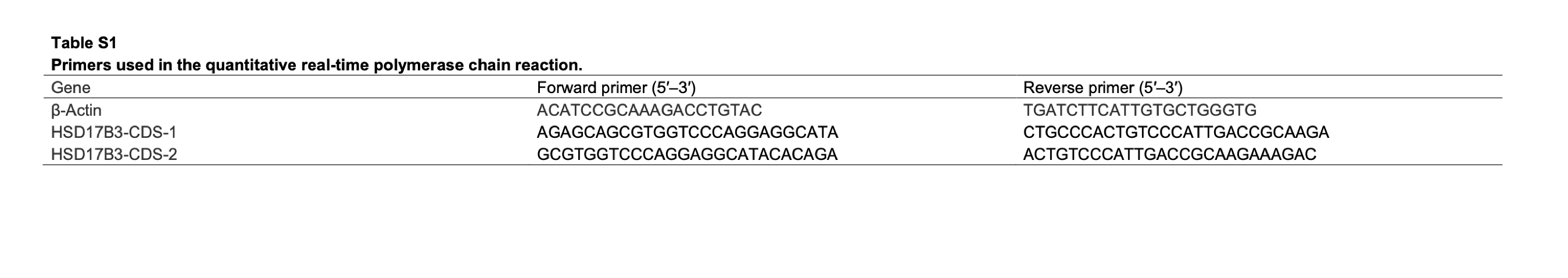

Supplement: Supplementary file 3 [file Table_1.docx]
